# Microbiological Characteristics of Pathogens Isolated From Blood Cultures of Patients With Acute Cholangitis: Insights From Patients With Biliary‐Enteric Anastomosis

**DOI:** 10.1002/jhbp.12193

**Published:** 2025-08-02

**Authors:** Yuta Kuhara, Hiroki Kitagawa, Yuki Kaiki, Keitaro Omori, Norifumi Shigemoto, Tomoyuki Akita, Kenichiro Uemura, Shingo Fukuma, Shinya Takahashi, Hiroki Ohge

**Affiliations:** ^1^ Department of Infectious Diseases Hiroshima University Hospital Hiroshima Japan; ^2^ Department of Surgery, Graduate School of Biomedical and Health Sciences Hiroshima University Hiroshima Japan; ^3^ Translational Research Center Hiroshima University Hiroshima Japan; ^4^ Department of Epidemiology, Disease Control and Prevention, Graduate School of Biomedical and Health Sciences Hiroshima University Hiroshima Japan

**Keywords:** ampC beta‐lactamase, blood culture, cholangitis, enterobacterales, pancreaticoduodenectomy

## Abstract

**Background:**

We aimed to investigate the microbiological characteristics of pathogens isolated from blood cultures (BCs) of patients with acute cholangitis (AC) after biliary‐enteric anastomosis and biliary intervention (BI).

**Methods:**

A retrospective analysis was conducted on 366 patients with AC and bacteremia between 2015 and 2024 at Hiroshima University Hospital. Patients were categorized into three groups: post‐biliary reconstruction‐associated AC (PBR‐AC), BI‐associated AC (BI‐AC), and common AC (C‐AC). Patients' clinical and microbiological data were statistically analyzed in each group.

**Results:**

The most frequently isolated pathogens were *Escherichia coli* and *Klebsiella* spp., accounting for > 50% of isolates in all groups. ESCPM spp. (*Enterobacter* spp., including *Klebsiella aerogenes* , *Serratia marcescens* , *Citrobacter freundii complex*, *Providencia* spp., and *Morganella morganii* ) were significantly associated with PBR‐ and BI‐AC compared with C‐AC. No significant differences in the prevalence of anaerobic bacteria were observed among the groups.

**Conclusions:**

The prevalence of ESCPM spp. isolated from BCs was significantly higher in the PBR‐ and BI‐AC groups than in the C‐AC group. The presence of biliary‐enteric anastomosis or BI history should be checked when determining the treatment strategy for AC. Cefepime may be a better antibiotic option for PBR‐ and BI‐AC, particularly in severe cases.

## Graphical abstract

Kuhara and colleagues analyzed 366 cases of bacteremic acute cholangitis, focusing on pathogens isolated from blood cultures. Escherichia coli and *Klebsiella* spp. predominated overall, whereas ESCPM organisms were significantly more frequent in patients with prior biliary‐enteric anastomosis or intervention, highlighting the importance of considering biliary history when selecting empirical antimicrobial therapy.

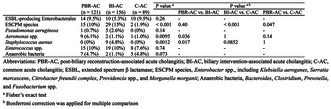

## 1 Introduction

Acute cholangitis (AC) remains a life‐threatening infection if not promptly and appropriately treated, with the mortality rate being as high as 2.1%–14% [1, 2]. The reported rates of bacteremia among patients with AC range from 21% to 71% [3]. Bacteremia in patients with AC results from a combination of increased bile duct pressure and bile colonization [4], followed by bacterial reflux into the hepatic veins and perihepatic lymphatics [5].

AC after biliary‐enteric anastomosis is a major complication after hepatobiliary‐pancreatic surgery, such as pancreaticoduodenectomy [6]. The exact mechanism underlying AC after biliary‐enteric anastomosis remains unclear; however, the most widely accepted explanation is that the loss of duodenal papilla function allows intestinal contents to reflux easily into the biliary duct, increasing the risk of retrograde biliary infections [5]. Previous studies have reported frequencies of postoperative AC after pancreaticoduodenectomy ranging from 4.8% to 19% [7]. AC after biliary‐enteric anastomosis negatively impacts quality of life; thus, administering appropriate antibiotic therapy is essential.

International practice guidelines for AC were developed and released in 2018 and referred to as Tokyo Guidelines 2018 (TG18). TG18 recommends the use of antibiotics for community‐acquired AC based on the severity grade and for healthcare‐associated AC [8]. It also recommends the use of anaerobic therapy for AC after biliary‐enteric anastomosis [1]. However, the microbiological characteristics of AC after biliary‐enteric anastomosis and stent‐related AC have not been fully described in TG18. Studies particularly focusing on the pathogens associated with AC after biliary‐enteric anastomosis are also limited [9, 10, 11]. In the present study, we aimed to investigate the microbiological characteristics of pathogens isolated from blood cultures (BCs) of patients with AC after biliary‐enteric anastomosis and stent‐related AC.

## 2 Methods

### 2.1 Patients

We retrospectively reviewed patients with AC and bacteremia treated at Hiroshima University Hospital between January 2015 and December 2024. The inclusion criteria were: (1) ≥ 18 years of age, (2) meeting the diagnostic criteria of AC based on TG18 [12], and (3) being BC‐positive. Cases of AC after biliary‐enteric anastomosis included both “definite” and “suspected” cases of TG18, whereas other cases of AC included “definite” cases only. The exclusion criteria were: (1) Occurrence of AC within 24 h of endoscopic retrograde cholangiopancreatography (ERCP), (2) occurrence of AC with external biliary drainage, such as endoscopic nasobiliary drainage (ENBD) or percutaneous transhepatic biliary drainage (PTBD), (3) occurrence of AC during the postoperative hospital stay after biliary reconstruction procedure apart from earlier postoperative complications, (4) occurrence of AC during antibiotic therapy, (5) BC results determined to be contaminated by the infectious diseases physicians, and (6) case of recurrent AC with bacteremia.

### 2.2 Definitions

We defined AC after biliary‐enteric anastomosis as post‐biliary reconstruction‐associated AC (PBR‐AC), AC with biliary interventions (BIs), such as internal biliary stents or endoscopic sphincterotomy (EST), as BI‐AC, and AC without biliary reconstruction or any BI as common AC (C‐AC).

Anastomotic stenosis was diagnosed based on definitive imaging findings, such as computed tomography (CT), magnetic resonance imaging, direct cholangiography, or endoscopy. The presence or absence of anastomotic stenosis was comprehensively assessed by clinicians who diagnosed AC, as previously described [13]. The cause of anastomotic stenosis was classified as either tumor‐related or unknown. Tumor‐related stenosis was diagnosed as local recurrence or peritoneal dissemination based on CT findings or endoscopic pathology, whereas unknown stenosis was defined as a stricture without an apparent cause.

The obstructed stent was comprehensively evaluated. Obstructed‐stent AC was defined by endoscopists as the absence of bile from the stent due to biliary sludge, food residue, or malignant ingrowth, whereas open‐stent AC was defined as the presence of bile from the stent or no BI such as ERCP or PTBD following AC, as previously described [13]. Healthcare‐associated AC and septic shock were defined as previously described [14, 15], respectively. Typical CT images of AC with a transient hepatic attenuation difference and pneumobilia were defined as previously described [13].

### 2.3 Data Collection

All clinical data, including demographic data, etiologies of AC, clinical symptoms, presence of septic shock, history of hepatobiliary‐pancreatic malignancy, microbiological results of BCs, severity grade based on TG18, complications such as liver abscess, acute pancreatitis, and endocarditis, and 30‐day mortality rate, were collected. Detailed data on the primary disease requiring the surgical procedure of biliary‐enteric anastomosis, information on CT scans, and hospitalization of patients with PBR‐AC were also obtained.

### 2.4 Microbiological Analysis

BC was performed using BacT/ALERT 3D (bioMérieux, Marcy l'Étoile, France) from January 1, 2015 to April 17, 2022, and BacT/ALERT Virtuo (bioMérieux) from April 18, 2022 to December 31, 2024. BacT/ALERT FA and FN Plus bottles (bioMérieux) were used during both periods. BCs were incubated for a maximum of 7 days. The bacterial species was identified using the Vitek 2 compact system (bioMérieux) from January 1, 2015 to March 31, 2021, and matrix‐assisted laser desorption/ionization (MALDI) time‐of‐flight mass spectrometry using a MALDI Biotyper Sirius system (Bruker Daltonik GmbH, Bremen, Germany) from April 1, 2021 to December 31, 2024. Extended‐spectrum β‐lactamase production was screened and confirmed using the disk diffusion methods as described in CLSI M100‐Ed34 [16].

### 2.5 Statistical Analyses

Categorical data were expressed as frequencies and proportions (%), whereas continuous variables were presented as medians and interquartile ranges. All categorical variables were compared using the Fisher's exact test, whereas continuous variables were compared using the Kruskal–Wallis test. For multiple comparisons, *p*‐values were adjusted using Bonferroni correction. Statistical significance was set at *p* < 0.05. Data analysis was performed using the JMP Pro software (version 18.0; SAS Institute Inc., Cary, NC, USA).

### 2.6 Ethics Statement

The study protocol was approved by the Ethics Committee for Epidemiology at Hiroshima University (No. E‐2133). The requirement for written informed consent was waived due to the retrospective design of the study.

## 3 Results

**FIGURE 1:**
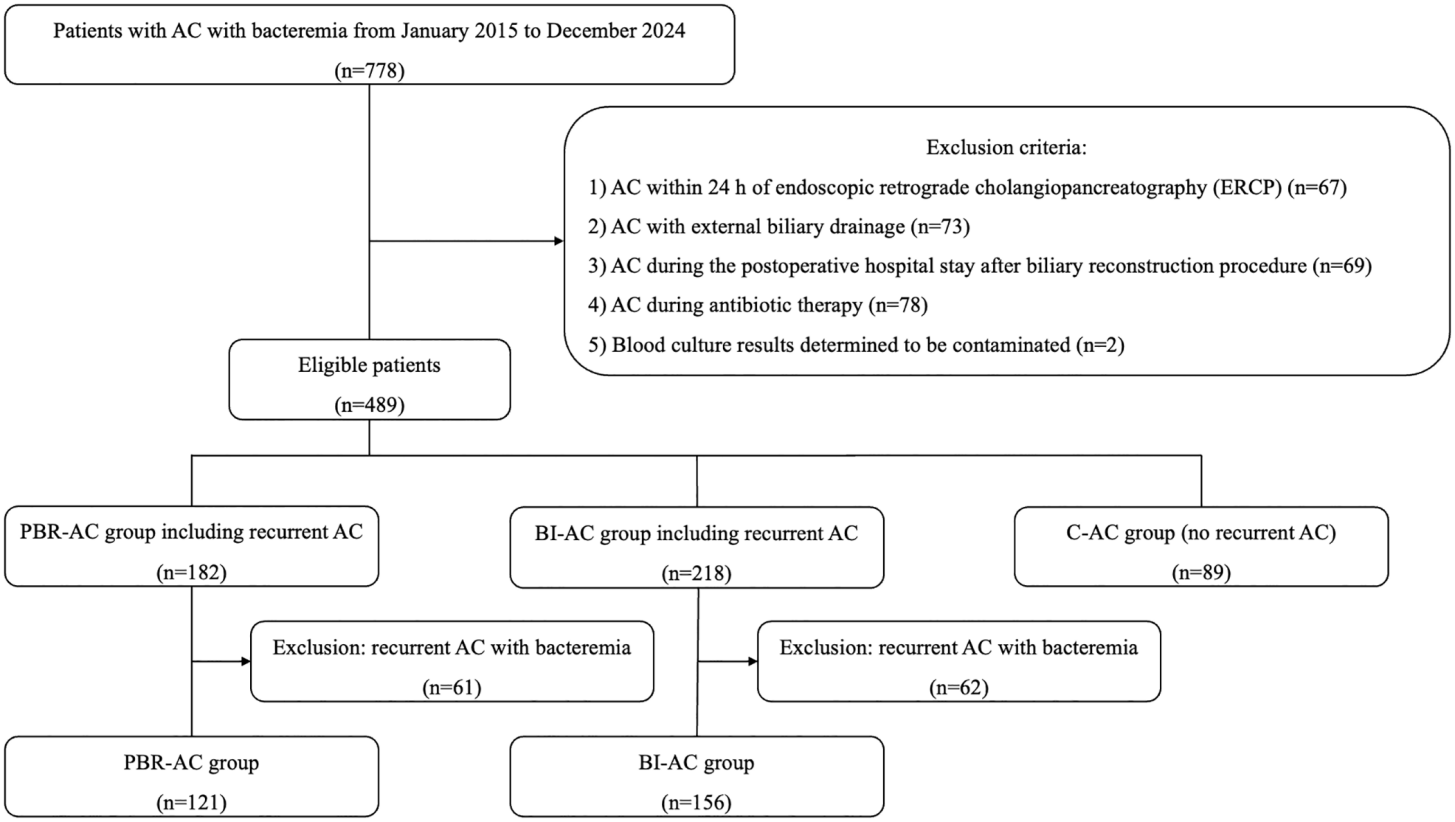
Flow diagram of patient selection according to the presence or absence of biliary reconstruction and biliary intervention. AC, acute cholangitis; BI‐AC, biliary intervention acute cholangitis; C‐AC, common acute cholangitis; PBR‐AC, post‐biliary reconstruction associated acute cholangitis.

A total of 778 patients with AC and bacteremia were identified, of whom 366 met the inclusion criteria after 412 were excluded. PBR‐, BI‐, and C‐AC accounted for 121 (33%), 156 (43%), and 89 (24%) cases, respectively (Figure 1). Table 1 presents the clinical characteristics of patients with AC and bacteremia. The most common etiologies of AC were unknown, stent‐related, and bile duct stones in the PBR‐AC (53%), BI‐AC (77%), and C‐AC (80%) groups, respectively. The rate of healthcare‐associated AC was significantly higher in the PBR‐AC and BI‐AC groups than in the C‐AC group. Based on the severity grades of TG18, 45%, 23%, and 31% of cases were classified as grades I, II, and III, respectively, with no significant differences observed among the groups. Liver abscess, a complication associated with AC, was observed in 13% of all cases, with the highest incidence observed in the PBR‐AC group (23%, *p* < 0.001).

**TABLE 1:** Clinical characteristics of patients with acute cholangitis and bacteremia.

	PBR‐AC	BI‐AC	C‐AC	Total
	(*n* = 121)	(*n* = 156)	(*n* = 89)	(*n* = 366)
Age (years), median (IQR)	74 (67–78)	72 (63–80)	77 (68–82)	74 (66–80)
Sex (%)				
Male	82 (68%)	99 (63%)	63 (71%)	244 (67%)
Polymicrobial bacteremia	21 (17%)	26 (17%)	12 (13%)	59 (16%)
Symptoms				
Fever	107 (88%)	137 (88%)	69 (78%)	313 (86%)
Abdominal pain	17 (14%)	60 (38%)	52 (58%)	129 (35%)
Septic shock	13 (11%)	18 (12%)	23 (26%)	54 (15%)
Treatment in ICU	13 (11%)	10 (6.4%)	20 (22%)	43 (12%)
Healthcare‐associated infection	97 (80%)	144 (92%)	59 (66%)	300 (82%)
History of biliary interventions				
Stent	19 (16%)	129 (83%)	0 (0%)	148 (40%)
EST without stent	0 (0%)	27 (17%)	0 (0%)	27 (7.4%)
History of malignancy				
Pancreas cancer	26 (21%)	47 (30%)	2 (2.2%)	75 (20%)
Biliary cancer	62 (51%)	54 (35%)	2 (2.2%)	118 (32%)
Liver cancer	6 (5.0%)	11 (7.1%)	9 (10%)	26 (7.1%)
Etiology of acute cholangitis				
Anastomotic stenosis	33 (27%)	0 (0%)	0 (0%)	33 (9.0%)
Cause of anastomotic stenosis: Tumor‐related	15 (12%)			15 (4.1%)
Cause of anastomotic stenosis: Unknown	18 (15%)			18 (4.9%)
Bile duct stone	10 (8.3%)	18 (12%)	71 (80%)	99 (27%)
Stent‐related	13 (11%)	120 (77%)	0 (0%)	133 (36%)
Open‐stent	7 (5.8%)	53 (34%)		60 (16%)
Obstructed‐stent	6 (5.0%)	67 (43%)		73 (20%)
Tumor	1 (0.8%)	9 (5.8%)	9 (10%)	19 (5.2%)
Unknown	64 (53%)	0 (0%)	7 (7.9%)	71 (19%)
Others	0 (0%)	9 (5.8%)	2 (2.2%)	11 (3.0%)
Diagnostic criteria by TG18				
Definite	75 (62%)	156 (100%)	89 (100%)	320 (87%)
Suspected	46 (38%)	0 (0%)	0 (0%)	46 (13%)
TG 18 severity grade (all cases)				
Grade I	51 (42%)	79 (51%)	35 (39%)	165 (45%)
Grade II	30 (25%)	38 (24%)	18 (20%)	86 (23%)
Grade III	40 (33%)	39 (25%)	36 (40%)	115 (31%)
TG 18 severity grade (community‐acquired cases)	24 (20%)	12 (7.7%)	30 (34%)	66 (18%)
Grade I	7 (5.8%)	8 (5.1%)	15 (17%)	30 (8.2%)
Grade II	9 (7.4%)	3 (1.9%)	4 (4.5%)	16 (4.4%)
Grade III	8 (6.6%)	1 (0.6%)	11 (12%)	20 (5.5%)
Biliary intervention after hospitalization	40 (33%)	119 (76%)	74 (83%)	233 (64%)
Complications				
Liver abscess	28 (23%)	12 (7.7%)	8 (9.0%)	48 (13%)
Acute pancreatitis	0 (0%)	2 (1.3%)	12 (13%)	14 (3.8%)
Endocarditis	0 (0%)	0 (0%)	0 (0%)	0 (0%)
30‐day all‐cause mortality rate	5 (4.1%)	10 (6.4%)	4 (4.5%)	19 (5.2%)
Cholangitis cause	1 (0.8%)	1 (0.6%)	3 (3.4%)	5 (1.4%)
Tumor‐related cause	4 (3.3%)	9 (5.8%)	1 (1.1%)	14 (3.8%)

Abbreviations: BI‐AC, biliary intervention‐associated acute cholangitis; C‐AC, common acute cholangitis; EST, endoscopic sphincterotomy; ICU, intensive care unit; IQR, interquartile range; PBR‐AC, post‐biliary reconstruction‐associated acute cholangitis; TG 18, Tokyo Guidelines 2018.

Table 2 presents the PBR‐AC cases, with 38% (46/121) being classified as “suspected” AC due to either the absence of characteristic imaging findings (*n* = 21) or lack of imaging studies, such as CT (*n* = 25).

**TABLE 2:** Clinical characteristics of PBR‐AC cases.

	PBR‐AC
	(*n* = 121)
Primary diseases requiring biliary‐enteric anastomosis	
Pancreatic carcinoma	26 (21%)
Perihilar bile duct carcinoma	29 (24%)
Distal bile duct carcinoma	10 (8.3%)
Gallbladder carcinoma	6 (5.0%)
Ampullary carcinoma	5 (4.1%)
Duodenal carcinoma	3 (2.5%)
Intrahepatic cholangiocarcinoma	10 (8.3%)
Liver carcinoma	6 (5.0%)
Benign disease	20 (17%)
Others or Unknown	6 (5.0%)
Surgical procedure	
Pancreaticoduodenectomy	57 (47%)
Hepatectomy with extrahepatic bile duct resection	38 (31%)
Extrahepatic bile duct resection alone	12 (9.9%)
Hepatopancreatoduodenectomy	7 (5.8%)
Liver transplantation	4 (3.3%)
Others or Unknown	3 (2.5%)
The cause of suspicious diagnosis of cholangitis	
Suspected cholangitis	46 (38%)
No presence of characteristic imaging findings based on TG18 criteria	21 (17%)
No images	25 (21%)
Dynamic CT	72 (60%)
Typical CT images	
Pneumobilia	32 (26%)
THAD	55 (45%)
Hospitalization	93 (77%)

Abbreviations: CT, computed tomography; PBR‐AC, post‐biliary reconstruction‐associated acute cholangitis; TG18, Tokyo Guidelines 2018; THAD, transient hepatic attenuation difference.

Table 3 presents the pathogens isolated from BCs in each group. *Escherichia coli* and *Klebsiella* spp. were the most frequently isolated pathogens, accounting for > 50% of cases across all groups and approximately 80% in the C‐AC group. The frequency of *Enterobacter cloacae complex* was 5.4%, 11%, and 1.9% in the PBR‐, BI‐, and C‐AC groups, respectively. *Staphylococcus aureus* (*n* = 9) was detected only in the BI‐AC group, primarily in the stent‐related cases (78%, 7/9). *Enterococcus* spp. was found in 10%, 10%, and 7.6% of cases in the PBR‐, BI‐, and C‐AC groups, respectively.

**TABLE 3:** Pathogens isolated from blood cultures of patients with acute cholangitis.

PBR‐AC		BI‐AC		C‐AC	
(*n* = 121)		(*n* = 156)		(*n* = 89)	
*Escherichia coli*	57 (36%)	*Klebsiella* spp.	58 (31%)	*Escherichia coli*	50 (48%)
ESBL−	43 (29%)	*K. pneumoniae*	30 (16%)	ESBL−	41 (39%)
ESBL+	14 (9.5%)	ESBL−	30 (16%)	ESBL+	9 (8.6%)
*Klebsiella* spp.	35 (29%)	ESBL+	0 (0%)	*Klebsiella* spp.	31 (30%)
*K. pneumoniae*	23 (16%)	*K. oxytoca*	13 (6.9%)	*K. pneumoniae*	22 (21%)
ESBL−	23 (16%)	*K. variicola*	8 (4.2%)	ESBL−	21 (20%)
ESBL+	0 (0%)	*K. aerogenes*	5 (2.6%)	ESBL+	1 (1.0%)
*K. oxytoca*	6 (4.1%)	*K. ozaenae*	2 (1.1%)	*K. oxytoca*	5 (4.8%)
*K. aerogenes*	3 (2.0%)	*Escherichia coli*	43 (23%)	*K. variicola*	4 (3.8%)
*K. variicola*	3 (2.0%)	ESBL−	33 (18%)	*Enterococcus* spp.	8 (7.6%)
*Enterococcus* spp.	15 (10%)	ESBL+	10 (5.3%)	*E. faecium*	3 (2.9%)
*E. faecium*	6 (4.1%)	*Enterobacter cloacae* complex	21 (11%)	*E. faecalis*	2 (1.9%)
*E. faecalis*	2 (1.4%)	*Enterococcus* spp.	19 (10%)	*E. gallinarum*	1 (1.0%)
*E. casseliflavus*	6 (4.1%)	*E. faecium*	8 (4.2%)	*E. avium*	1 (1.0%)
*E. gallinarum*	1 (0.7%)	*E. faecalis*	9 (4.8%)	Other *Enterococcus*	1 (1.0%)
*Aeromonas* spp.	9 (6.1%)	*E. raffinosus*	1 (0.5%)	*Streptococcus* spp.	4 (3.8%)
*Enterobacter cloacae* complex	8 (5.4%)	*E. casseliflavus*	1 (0.5%)	*Bacteroides fragilis*	3 (2.9%)
*Streptococcus* spp.	7 (4.7%)	*Staphylococcus aureus*	9 (4.8%)	*Clostridium perfringens*	2 (1.9%)
*Clostridium perfringens*	5 (3.4%)	MSSA	7 (3.7%)	*Enterobacter cloacae* complex	2 (1.9%)
*Edwardsiella tarda*	3 (2.0%)	MRSA	2 (1.1%)	*Edwardsiella tarda*	2 (1.9%)
*Citrobacter freundii*	2 (1.4%)	*Streptococcus* spp.	7 (3.7%)	*Acinetobacter baumannii*	1 (1.0%)
*Acinetobacter baumannii*	2 (1.4%)	*Pseudomonas aeruginosa*	5 (2.6%)	*Aeromonas* spp.	1 (1.0%)
*Fusobacterium nucleatum*	1 (0.7%)	*Edwardsiella tarda*	4 (2.1%)	*Vibrio fluvialis*	1 (1.0%)
*Morganella morganii*	1 (0.7%)	*Acinetobacter baumannii*	4 (2.1%)		
*Prevotella buccae*	1 (0.7%)	*Citrobacter* spp.	4 (2.1%)		
*Serratia marcescens*	1 (0.7%)	*C. koseri*	3 (1.6%)		
*Pseudomonas aeruginosa*	1 (0.7%)	*C. freundii*	1 (0.5%)		
		*Aeromonas* spp.	2 (1.1%)		
		*Proteus* spp.	2 (1.1%)		
		*Serratia marcescens*	2 (1.1%)		
		*Clostridium perfringens*	1 (0.5%)		
		*Neisseria subflava*	1 (0.5%)		
		*Pantoea* spp.	1 (0.5%)		
		*Pasteurella multocida*	1 (0.5%)		
		*Prevotella buccae*	1 (0.5%)		
		*Raoultella ornithinolytica*	1 (0.5%)		
		*Stenotrophomonas maltophilia*	1 (0.5%)		
		*Kosakonia radicincitans*	1 (0.5%)		
		Other	1 (0.5%)		
Total	148	Total	189	Total	105

Abbreviations: BI‐AC, biliary intervention‐associated acute cholangitis; C‐AC, common acute cholangitis; ESBL, extended spectrum β‐lactamase; MRSA, methicillin‐resistant *Staphylococcus aureus* ; MSSA, methicillin‐susceptible *Staphylococcus aureus* ; PBR‐AC, post‐biliary reconstruction‐associated acute cholangitis.

Table 4 presents the comparison of pathogen distribution among the three groups. The prevalence of ESCPM spp. (*Enterobacter* spp., including *Klebsiella aerogenes* , *Serratia marcescens* , *Citrobacter freundii complex*, *Providencia* spp., and *Morganella morganii* ) was significantly higher in the PBR‐ and BI‐AC groups than in the C‐AC group (PBA‐AC vs. C‐AC: 10% vs. 1.9%, *p* = 0.047; BI‐AC vs. C‐AC: 15% vs. 1.9%, *p* < 0.001). Anaerobes, including *Bacteroides*, *Clostridium*, *Prevotella*, and *Fusobacterium* spp., showed no significant differences among the groups. The prevalence of *Aeromonas* spp. was higher in the PBR‐AC (6.1%) than in the BI‐ and C‐AC groups.

**TABLE 4:** Comparison of pathogen distribution among the three groups.

	PBR‐AC	BI‐AC	C‐AC	*p* ^a^	*p* ^a^, ^b^		
	(*n* = 121)	(*n* = 156)	(*n* = 89)		PBR‐AC vs. BI‐AC	BI‐AC vs. C‐AC	PBR‐AC vs. C‐AC
ESBL‐producing Enterobacterales	14 (9.5%)	10 (5.3%)	10 (9.5%)	0.26	—	—	—
ESCPM species	15 (10%)	29 (15%)	2 (1.9%)	< 0.001	0.40	< 0.001	0.047
*Pseudomonas aeruginosa*	1 (0.7%)	5 (2.6%)	0 (0%)	0.14	—	—	—
*Aeromonas* spp.	9 (6.1%)	2 (1.1%)	1 (1.0%)	0.0095	0.036	1	0.14
*Staphylococcus aureus*	0 (0%)	9 (4.8%)	0 (0%)	0.0012	0.017	0.0852	1
*Enterococcus* spp.	15 (10%)	19 (10%)	8 (7.6%)	0.74	—	—	—
Anaerobic bacteria	7 (4.7%)	2 (1.1%)	5 (4.8%)	0.073	—	—	—

*Note:* ESCPM species, *Enterobacter* spp., including *Klebsiella aerogenes* , *Serratia marcescens* , *Citrobacter freundii complex*, *Providencia* spp., and *Morganella morganii* ; Anaerobic bacteria, *Bacteroides*, *Clostridium*, *Prevotella*, and *Fusobacterium* spp.

Abbreviations: BI‐AC, biliary intervention‐associated acute cholangitis; C‐AC, common acute cholangitis; ESBL, extended spectrum β lactamase; PBR‐AC, post‐biliary reconstruction‐associated acute cholangitis.

^a^ Fisher's exact test.

^b^ Bonferroni correction was applied for multiple comparisons.

Tables 5 and 6 present the pathogen distribution in healthcare‐associated AC cases. The findings for healthcare‐associated AC mirrored those of the overall cohort, with ESCPM spp. being more frequently isolated in the PBR‐ and BI‐AC groups than in the C‐AC group (PBA‐AC vs. C‐AC: 12% vs. 1.4%, *p* = 0.029; BI‐AC vs. C‐AC: 16% vs. 1.4%, *p* < 0.001).

**TABLE 5:** Pathogens isolated from blood cultures of patients with healthcare‐associated acute cholangitis.

PBR‐AC		BI‐AC		C‐AC	
(*n* = 97)		(*n* = 144)		(*n* = 59)	
*Escherichia coli*	47 (38%)	*Klebsiella* spp.	56 (32%)	*Escherichia coli*	36 (51%)
ESBL−	34 (28%)	*K. pneumoniae*	29 (17%)	ESBL−	29 (41%)
ESBL+	13 (11%)	ESBL−	29 (17%)	ESBL+	7 (10%)
*Klebsiella* spp.	32 (26%)	ESBL+	0 (0%)	*Klebsiella* spp.	21 (30%)
*K. pneumoniae*	21 (17%)	*K. oxytoca*	12 (6.8%)	*K. pneumoniae*	16 (23%)
ESBL−	21 (17%)	*K. variicola*	8 (4.5%)	ESBL−	15 (21%)
ESBL+	0 (0%)	*K. aerogenes*	5 (2.8%)	ESBL+	1 (1.4%)
*K. oxytoca*	6 (4.9%)	*K. ozaenae*	2 (1.1%)	*K. oxytoca*	3 (4.2%)
*K. aerogenes*	3 (2.4%)	*Escherichia coli*	39 (22%)	*K. variicola*	2 (2.8%)
*K. variicola*	2 (1.6%)	ESBL−	31 (18%)	*Enterococcus* spp.	5 (7.0%)
*Enterococcus* spp.	10 (8.1%)	ESBL+	8 (4.5%)	*E. faecium*	3 (4.2%)
*E. faecium*	4 (3.3%)	*Enterobacter cloacae* complex	21 (12%)	*E. faecalis*	2 (2.8%)
*E. faecalis*	2 (1.6%)	*Enterococcus* spp.	15 (8.5%)	*Bacteroides fragilis*	3 (4.2%)
*E. casseliflavus*	3 (2.4%)	*E. faecium*	6 (3.4%)	*Streptococcus* spp.	1 (1.4%)
*E. gallinarum*	1 (0.8%)	*E. faecalis*	8 (4.5%)	*Clostridium perfringens*	1 (1.4%)
*Enterobacter cloacae* complex	8 (6.5%)	*E. raffinosus*	1 (0.6%)	*Enterobacter cloacae* complex	1 (1.4%)
*Aeromonas* spp.	6 (4.9%)	*Staphylococcus aureus*	9 (5.1%)	*Acinetobacter baumannii*	1 (1.4%)
*Streptococcus* spp.	5 (4.1%)	MSSA	7 (4.0%)	*Vibrio fluvialis*	1 (1.4%)
*Clostridium perfringens*	5 (4.1%)	MRSA	2 (1.1%)	*Edwardsiella tarda*	1 (1.4%)
*Citrobacter freundii*	2 (1.6%)	*Streptococcus* spp.	8 (4.5%)		
*Acinetobacter baumannii*	2 (1.6%)	*Pseudomonas aeruginosa*	4 (2.3%)		
*Edwardsiella tarda*	1 (0.8%)	*Edwardsiella tarda*	3 (1.7%)		
*Fusobacterium nucleatum*	1 (0.8%)	*Acinetobacter baumannii*	3 (1.7%)		
*Morganella morganii*	1 (0.8%)	*Citrobacter* spp.	3 (1.7%)		
*Prevotella buccae*	1 (0.8%)	*C. koseri*	2 (1.1%)		
*Serratia marcescens*	1 (0.8%)	*C. freundii*	1 (0.6%)		
*Pseudomonas aeruginosa*	1 (0.8%)	*Aeromonas* spp.	2 (1.1%)		
		*Proteus* spp.	2 (1.1%)		
		*Serratia marcescens*	2 (1.1%)		
		*Clostridium perfringens*	1 (0.6%)		
		*Neisseria subflava*	1 (0.6%)		
		*Pantoea* spp.	1 (0.6%)		
		*Gemella morbillorum*	1 (0.6%)		
		*Prevotella buccae*	1 (0.6%)		
		*Raoultella ornithinolytica*	1 (0.6%)		
		*Stenotrophomonas maltophilia*	1 (0.6%)		
		*Kosakonia radicincitans*	1 (0.6%)		
		Other	1 (0.6%)		
Total	123	Total	176	Total	71

Abbreviations: BI‐AC, biliary intervention‐associated acute cholangitis; C‐AC, common acute cholangitis; ESBL, extended spectrum β lactamase; MRSA, methicillin‐resistant *Staphylococcus aureus* ; MSSA, methicillin‐susceptible *Staphylococcus aureus* ; PBR‐AC, post‐biliary reconstruction‐associated acute cholangitis.

**TABLE 6:** Comparison of pathogen distribution with healthcare‐associated acute cholangitis among the three groups.

	PBR‐AC	BI‐AC	C‐AC	*p* ^a^	*p* ^a^, ^b^		
	(*n* = 97)	(*n* = 144)	(*n* = 59)		PBR‐AC vs. BI‐AC	BI‐AC vs. C‐AC	PBR‐AC vs. C‐AC
ESBL‐producing Enterobacterales	13 (11%)	8 (4.5%)	8 (11%)	0.069	—	—	—
ESCPM species	15 (12%)	29 (16%)	1 (1.4%)	0.0034	0.91	< 0.001	0.029
*Pseudomonas aeruginosa*	1 (0.8%)	4 (2.3%)	0 (0%)	0.51	—	—	—
*Aeromonas* spp.	6 (4.9%)	2 (1.1%)	0 (0%)	0.041	0.19	1	0.25
*Staphylococcus aureus*	0 (0%)	9 (5.1%)	0 (0%)	0.0063	0.037	0.18	1
*Enterococcus* spp.	10 (8.1%)	15 (8.5%)	5 (7.0%)	0.96	—	—	—
Anaerobic bacteria	7 (5.7%)	2 (1.1%)	4 (5.6%)	0.035	0.098	0.18	1

*Note:* ESCPM species, *Enterobacter* spp., including *Klebsiella aerogenes* , *Serratia marcescens* , *Citrobacter freundii complex*, *Providencia* spp., and *Morganella morganii* ; Anaerobic bacteria; *Bacteroides*, *Clostridium*, *Prevotella*, and *Fusobacterium* spp.

Abbreviations: BI‐AC, biliary intervention‐associated acute cholangitis; C‐AC, common acute cholangitis; ESBL, extended spectrum β lactamase; PBR‐AC, post‐biliary reconstruction‐associated acute cholangitis.

^a^ Fisher's exact test.

^b^ Bonferroni correction was applied for multiple comparisons.

## 4 Discussion

In this study, we investigated the characteristics of the microorganisms isolated from BCs of patients with AC, focusing on the history of biliary‐enteric anastomosis and BIs. *E. coli* and *K. pneumoniae* were the most frequently isolated organisms from BCs in all groups. This finding is similar to that shown in a previous study in which *E. coli* and *K. pneumoniae* were reported as the most frequently isolated organisms across all severity grades based on TG18 [1]. In the present study, the prevalence of ESCPM spp. isolated from BCs was significantly higher in the PBR‐ and BI‐AC groups than in the C‐AC group. Notably, the prevalence of ESCPM spp. isolated from BCs in the present study was 10% and 15% in the PBR‐ and BI‐AC groups, respectively, which were higher than those reported by Gomi et al., who found that the overall prevalence of ESCPM spp. isolated from BCs was 6.7%, with AC severity grades I, II, and III accounting for 7.5%, 5.2%, and 7.8%, respectively [1]. However, in the study by Gomi et al., the number of patients with a history of biliary‐enteric anastomosis or BIs was not reported. The high prevalence of ESCPM spp. isolated from BCs in the PBR‐ and BI‐AC groups may be due to similar anatomical features of loss of duodenal papilla function. Only a few studies have focused on the microbiological features of pathogens isolated from BCs of patients with PBR‐AC. The positive rate of the BCs of patients with PBR‐AC ranges from 36% to 67%, with *E. coli* being the most frequently isolated pathogen, followed by *E. faecium* and *K. pneumonia* [9, 10, 11]. One study showed that the proportion of *K. aerogenes* isolated from BCs of patients with PBR‐AC was 1.1% (1/93), with no other ESCPM spp. described [11]. Unlike the limited reports on PBR‐AC, there have been several reports on BI‐AC. The presence of biliary stents or EST has been associated with an increased frequency of *Klebsiella* spp., *Pseudomonas aeruginosa* , *Enterobacter* spp., and *Enterococcus* spp. in bile cultures [17]. Other studies reported that the presence of biliary stents or EST was associated with an increased frequency of *Enterococcus* spp. in bile cultures and BCs [18, 19]. However, in the present study, the frequency of *Enterococcus* spp. isolated from BC was not significantly different among the groups. Among patients who underwent pancreaticoduodenectomy, ESCPM spp., *Klebsiella* spp., *P. aeruginosa* , and *Enterococcus* spp. were more frequently isolated from intraoperative bile cultures of those who underwent preoperative biliary drainage than in those who did not [20, 21]. Because these organisms colonize the biliary tract after stent insertion or EST, there may be an increased risk of them being the causative agents of cholangitis.

In a previous study, anaerobic pathogens, including *Bacteroides* spp., comprised only 1%–2% of the microorganisms isolated from blood and bile of patients with AC [1]. TG18 and the Infectious Diseases Society of America (IDSA) guideline recommend that patients who undergo biliary‐enteric anastomosis should be treated for anaerobic pathogens [8, 14]. However, no significant differences in the isolation of anaerobic bacteria were observed between the PBR‐ and C‐AC groups in the present study. In addition, *Bacteroides fragilis* was not isolated from BCs in the PBR‐AC group. Obligate anaerobic bacteria, such as *B. fragilis* , which require long incubation times, may be underestimated if anaerobic bacteria are present in the BC bottle along with fast‐growing aerobic bacteria, such as *E. coli* and *K. pneumoniae* . Further studies regarding epidemiological information on whether biliary‐enteric anastomosis is a risk factor for anaerobic bacteria isolated from bile cultures and BCs of patients with AC are required.

*Aeromonas* spp. infections pose environmental risks and are typically isolated from water and food [22]. The prevalence of *Aeromonas* spp. isolated from BCs of patients with AC has been reported to be low (0.4%–3.4%) [1, 23], which is similar to our results in all AC cases (2.7%). A previous report showed that > 80% of AC cases caused by *Aeromonas* spp. were associated with prior exploration of the biliary tract [20]. In the present study, the prevalence of *Aeromonas* spp. isolated from BCs was higher in the PBR‐AC than in other groups. All patients with *Aeromonas* bacteremia in the PBR‐AC group underwent Roux‐en‐Y reconstruction. Food does not pass through the biliary‐enteric anastomosis in patients who undergo Roux‐en‐Y reconstruction; however, it is unclear why *Aeromonas* spp. were more frequently isolated from BCs in the PBR‐AC than from those in other groups.

Biliary tract infections caused by *S. aureus* are rare. In previous reports, *S. aureus* was isolated from only 0.9%–2.9% of bile cultures and 0%–2.3% of BCs of patients with biliary tract infections [1, 4, 24]. Our findings correspond with the results presented in these reports. However, all patients with *S. aureus* bacteremia, particularly those with stent‐related AC (78%, 7/9), were in the BI‐AC group. Yang et al. reported that 71% of patients with biliary infections presenting with *S. aureus* bacteremia had biliary drainage stents [24]. In a previous study, infective endocarditis was reported in 17 (0.3%) of 6433 patients with AC [1]. TG18 suggested that administering antimicrobial therapy for 2 weeks is prudent to decrease the risk of infective endocarditis in patients with AC and bacteremia caused by gram‐positive bacteria, such as *Enterococcus* spp. and *S. aureus* [11]. However, in the present study, no infective endocarditis cases were detected.

TG18 provided recommendations for antimicrobial therapy for community‐acquired AC based on the severity grade and for healthcare‐associated AC [11]. In the present study, the rate of healthcare‐associated AC was significantly higher in the PBR‐ and BI‐AC groups than in the C‐AC group. In contrast, no difference in the severity grade was observed among the groups. However, the prevalence of ESCPM spp. isolated from BCs was significantly higher in the PBR‐ and BI‐AC groups than in the C‐AC group. Similar results were observed for the healthcare‐associated AC cases. ESCPM spp. carry chromosomal AmpC β‐lactamases. *Enterobacter cloacae complex*, *Klebsiella* (formerly *Enterobacter*) *aerogenes*, and *Citrobacter freundii* complex are the most common Enterobacterales at moderate risk of clinically significant inducible AmpC production [25]. TG18 recommends the use of antimicrobial agents with anti‐pseudomonal activities for Grade III community‐acquired AC and healthcare‐associated AC [11]. *P. aeruginosa* is a known virulent pathogen, and failure to cover this organism empirically in critically ill patients may result in excess mortality. However, a recent study showed very few isolates, ranging from 1.1% to 3.1% among those from BCs and 2.5% to 3.6% among those from bile cultures obtained from patients with AC [1]. In the present study, *P. aeruginosa* was isolated from 0.7% and 2.6% of BCs in the PBR‐ and BI‐AC groups, respectively, but was not detected in the C‐AC group. Our results suggest that for PBR‐ and BI‐AC, antibiotic selection for ESCPM spp., as well as *E. coli* and *K. pneumoniae* , should be performed regardless of community‐acquired or healthcare‐associated AC. Therefore, based on IDSA guidance [25], cefepime may be a better antibiotic option for PBR‐ and BI‐AC, particularly in severe cases. Third‐generation cephalosporins such as ceftriaxone and piperacillin‐tazobactam may also be reasonable for non‐severe or source‐controlled cases. In addition, local antibiograms are essential for the timely selection of susceptible antibiotics in clinical settings. These results may be associated with those of recent reports suggesting that the use of third‐ or fourth‐generation cephalosporins and piperacillin‐tazobactam as perioperative prophylaxis for pancreatoduodenectomy reduces postoperative surgical site infections [26, 27, 28]. Further studies on the antimicrobial therapies for PBR‐ and BI‐AC are required.

The incidence of liver abscess after AC is reportedly 2.0% across all pathologies [1]. In cases of PBR‐AC in a previous study, the incidence of liver abscess was 4.3%, including cases of AC with negative BC [11]. Compared with that reported in these earlier studies, our study revealed a higher incidence of 13%, likely because we only included BC‐positive cases. Notably, the incidence of liver abscess in the PBR‐AC group (23%) was significantly higher than that in BI‐AC (7.7%) and C‐AC (9.0%) groups.

In the present study, “suspected” AC cases were included in the PBR‐AC group because the rate of “definite” diagnoses was significantly lower than that in the group of cases of AC without biliary‐enteric anastomosis, based on the diagnostic criteria provided in TG18 [13, 29]. Kato et al. suggested adding transient hepatic attenuation difference and pneumobilia to TG imaging criteria to improve TG diagnostic performance [13]. Following this criteria by Kato et al. the proportion of “definite” AC cases was increased from 62% (75/121) to 74% (89/121) in this study.

This study has some limitations. First, this was a retrospective, non‐randomized, single‐center study with inherent selection bias or data accuracy. Our hospital is a tertiary‐care hospital. Therefore, the number of patients in the C‐AC group was small, and the rate of healthcare‐associated AC in the C‐AC group was high (66%). Second, we excluded cases of recurrent AC and AC without bacteremia. Third, we investigated only the microbiological characteristics of pathogens isolated from BCs of patients with AC. Bile cultures were excluded from the endpoints because of the difficulty in collecting bile samples before administering antibiotics, particularly in PBR‐AC cases. Therefore, considering all the limitations, extrapolating the results of this study is challenging. Further large‐population studies are required to investigate the clinical and microbiological characteristics of PBR‐AC and BI‐AC compared with those of C‐AC.

In conclusion, the prevalence of ESCPM spp. isolated from BCs was significantly higher in the PBR‐ and BI‐AC groups than in the C‐AC group. The presence of biliary‐enteric anastomosis or BI history should be checked when determining the treatment strategy for AC. Cefepime may be a better antibiotic option for PBR‐ and BI‐AC, particularly in severe cases. Further studies on the antimicrobial therapies for PBR‐ and BI‐AC are required.

## Author Contributions

H.K. designed the study. Y.K., H.K., and Y.K. participated in data acquisition. Y.K., H.K., T.A., S.T., K.O., and N.S. analyzed and interpreted the data. Y.K. and H.K. drafted the manuscript. H.K., N.S., K.U., S.T., and H.O. critically revised the manuscript. All the authors approved the final version of the manuscript.

## Conflicts of Interest

The authors declare no conflicts of interest.

## Data Availability

The datasets used or analyzed during the current study are available from the corresponding author upon reasonable request.
